# Randomized, placebo-controlled, phase IV pilot study of ramosetron to evaluate the co-primary end points in male patients with irritable bowel syndrome with diarrhea

**DOI:** 10.1186/s13030-017-0093-9

**Published:** 2017-03-16

**Authors:** Motoko Ida, Akito Nishida, Hiraku Akiho, Yoshihiro Nakashima, Kei Matsueda, Shin Fukudo

**Affiliations:** 1 Japan-Asia Planning & Administration, Medical & Development, Astellas Pharma Inc., 2-5-1 Nihonbashi-Honcho, Chuo-ku, Tokyo, 103-8411 Japan; 2 Development Project Management, Astellas Pharma Inc., Tokyo, Japan; 3 Japan-Asia Clinical Development 2, Development, Astellas Pharma Inc., Tokyo, Japan; 4 Japan-Asia Data Science, Development, Astellas Pharma Inc., Tokyo, Japan; 5 Sakura Life Clinic, Tokyo, Japan; 6 Department of Behavioral Medicine, Tohoku University Graduate School of Medicine, Sendai, Japan

**Keywords:** 5-hydroxytryptamine (5-HT), Abdominal pain, Abdominal discomfort, Global improvement, Stool consistency

## Abstract

**Background:**

Global assessment allows patients to assess improvement in multiple irritable bowel syndrome (IBS) symptoms. However, it was deemed important to assess “clinically meaningful improvements, focusing on the patient’s chief complaint and the severity of major IBS symptoms” in addition to global assessment to show how ramosetron is effective for individual IBS symptoms. This is a pilot study to explore clinical endpoints focusing on the chief complaint of patients with IBS with diarrhea (IBS-D).

**Methods:**

The same database was used in a previously reported post-marketing phase IV, randomized placebo-controlled pilot trial in male patients with IBS-D. The hypothesis is completely different from that of the other study. Patients with IBS-D diagnosed according to Rome III criteria were given either 5 μg of ramosetron (*n* = 47) or placebo (*n* = 51) once daily for 12 weeks after a one-week baseline period. To explore and examine endpoints that allow evaluation of “clinically meaningful improvements focusing on the patient’s chief complaint,” the chief complaint and its relief by this study drug were assessed in this exploratory study.

**Results:**

Rates of patients with abdominal pain/discomfort, stool form and stool frequency which patients had as a chief complaint before administration were 34.0, 19.1 and 25.5%, respectively, in the ramosetron 5 μg group and 42.0, 18.0, and 20.0% in the placebo group. Responder rates for improvement in symptoms of the chief complaint that patients had before administration were 53.2% in the ramosetron 5 μg group and 42.0% in the placebo group at the last point. The greatest symptomatic improvement in the chief complaint in the ramosetron 5 μg group compared to the placebo group was shown with respect to stool consistency. Bristol Stool Form Scale (BSFS) scores were significantly lower in the ramosetron group than in the placebo group (4.36 ± 1.195 vs 4.85 ± 0.890 at the last point, *P* = 0.027) throughout the treatment period, except at week 6.

**Conclusions:**

Ramosetron acted most effectively on stool consistency. Improvement in stool consistency is considered to be a clinically meaningful endpoint in showing how ramosetron was effective for individual IBS symptoms. (Clinicaltrials.gov ID: NCT00918411. Registered 9 June 2009).

## Background

Irritable bowel syndrome (IBS) is a functional disease characterized by prolonged persistence or recurrence of abnormal bowel habits and abdominal pain and discomfort without organic diseases or biochemical abnormalities [1]. IBS is not a life-threatening disease, but has been shown to limit the activity of patients and to negatively impact social functioning, with substantial economic loss [2]. It is also reported that IBS can severely compromise the patient’s quality of life (QOL), even to a greater extent than in patients with end-stage renal disease or patients with diabetes [3]. Surveys conducted outside Japan have reported that the estimated prevalence of IBS in the general population is from 5 to 20%, with about 200 new patients per 100,000 population per year [4, 5]. In Japan, a large population-based internet survey using the Rome III criteria by Kanazawa et al. revealed that about 16.5% of the survey population met the Rome III criteria for IBS [6]. A web-based survey by Miwa that used Rome III showed that 13% of the respondents had IBS [7].

A variety of factors are considered to be involved in the etiology of IBS, including abnormal gastrointestinal (GI) motility, visceral hypersensitivity, abnormal brain–gut interactions, GI infection, and sociopsychological strain [8]. Stress-related disturbance of brain–gut interactions is a particularly important factor in functional gastrointestinal disorders including IBS [9]. IBS patients reported significantly more stress than controls without bowel dysfunction and the slope of the regression equation relating bowel symptoms to stress was significantly steeper for the IBS group [10]. Stress is therefore considered to induce abnormal GI motility via efferent nerves, which in turn worsen the stress response by stimulating afferent nerves, resulting visceral hypersensitivity. This vicious cycle is thought to cause persistence of bowel symptoms. Patients with IBS with diarrhea (IBS-D) show exaggerated colonic motility in response to colonic distention [11] and secretion of 5-hydroxytryptamine (5-HT, serotonin) [12].

Ramosetron, a potent and selective 5-hydroxytryptamine 3 (5-HT_3_) receptor antagonist [13–16], was developed and has been approved at a dosage of “ramosetron 5 μg for IBS-D in male patients” since July, 2008 in Japan [17, 18], followed by a supplemental indication and dosage of “ramosetron 2.5 μg for female patients” in May, 2015 [19–21]. In rats, ramosetron clearly reduces stress-induced diarrhea and defecation caused by corticotrophin-releasing hormone [14, 15]. In addition, ramosetron increases the threshold of abdominal pain responses induced by colonic distension in rats [22]. Thus, ramosetron is expected to improve IBS symptoms via reducing the vicious cycle for stress-related disturbance of brain–gut interactions.

The choice of primary endpoint for a clinical trial is one of the most important determinants of the ability of a clinical trial to demonstrate the efficacy of therapeutic agents. The Committee on Functional Bowel Disorders and Functional Abdominal Pain, an international working group for the establishment of diagnostic criteria for functional gastrointestinal tract disturbances including IBS, discussed primary variables in clinical studies. It was concluded that, since IBS is a syndrome, instead of evaluating individual symptoms the improvement of overall symptoms of the syndrome should be assessed and subjects should evaluate the effects of therapeutics because improvement of subjective symptoms is clinically important for IBS [23]. Consequently, the global assessment of relief of overall IBS symptoms was chosen to be the primary variable for previous clinical studies of ramosetron [17, 18], and its efficacy was demonstrated. Global assessment allows patients to assess improvement of multiple IBS symptoms, however, global assessment cannot show how ramosetron is effective for individual IBS symptoms. Therefore, it was deemed beneficial to assess “clinically meaningful improvements, focusing on the patient’s chief complaint and the severity of major IBS symptoms” in addition to the global assessment. This study was conducted as a pilot study to explore and examine those variables. Results that focus on the severity of major IBS symptoms are contained in a previous report [24]. We thus used the same database as the previous report, but the hypothesis was completely different from that of the previous study. This report concentrates on improvement of the patient’s chief complaint in a clinical study of ramosetron.

## Methods

### Patient population

The patients were the same as in our previous report [24]. In brief, this study was conducted from June 2009 to December 2009 at 25 Japanese centers that have departments of gastroenterology. Almost all of the sites were primary care. Male outpatients aged 20–64 years were diagnosed with IBS-D based on the Rome III criteria. The study protocol was designed in accordance with the Declaration of Helsinki and was approved by the institutional review board at each site. All patients provided written informed consent prior to participating in study-related procedures.

Patients satisfying the inclusion and exclusion criteria were enrolled in this study. Based on a medical interview conducted by the attending physician before provisional registration, patients were excluded if any of the following were evident: a history of resection of the stomach, small intestine, or large intestine (excluding appendicitis or resection of benign polyps); history or current evidence of inflammatory bowel disease; history or current evidence of ischemic colitis, concurrent infectious enteritis, hyperthyroidism, hypothyroidism, or other diseases that may affect gastrointestinal transit or colonic function; history or current evidence of abuse of drugs or alcohol within the previous year; malignant tumors; current evidence of severe depression or a severe anxiety disorder that could potentially affect the evaluation of study drug efficacy; concurrent serious cardiovascular, respiratory, renal, hepatic, gastrointestinal (excluding IBS), hematological, or neurological/psychiatric diseases; or a history of drug allergies. Other inclusion and exclusion criteria can be found in our previous report [24].

### Study design

This randomized, placebo-controlled, pilot clinical trial comprised a provisional registration period, a one-week baseline period, and a 12-week treatment period, similar to previous studies [17, 18]. Following the baseline period, eligible patients were randomly assigned to 12-week oral treatment with placebo or ramosetron hydrochloride 5 μg once daily before breakfast. Visits were scheduled at Weeks 2, 4, 8, and 12 (or at discontinuation) to assess treatment efficacy, drug compliance, and occurrence of adverse events. Randomization was performed in a 1:1 ratio using a block size of four based on a randomization list developed by a third-party contract research organization. Placebo tablets were externally distinguishable from ramosetron hydrochloride tablets, however, they were indistinguishable when packaged in press through pack sheets. All patients, investigators, and sponsors were blinded until all observations and evaluations were completed, statistical analysis plans were finalized, and all data had been locked. All authors had access to the study data and reviewed and approved the final manuscript.

### Data collection

Patients recorded Bristol Stool Form Scale (BSFS) [25] for every bowel movement throughout the study period in their diaries. They scored the severity of all abdominal pain/discomfort they had experienced on a five-point ordinate (numerical rating) scale and their continuous time at baseline, from week 1 to week 4, and at weeks 8 and 12. Every seven days during the treatment period, patients also graded summarized IBS symptoms compared with the baseline period on a five-point ordinate scale as follows: relief from overall IBS symptoms and abdominal pain/discomfort (0, completely relieved; 1, considerably relieved; 2, somewhat relieved; 3, unchanged; and 4, worsened) and improvement in abnormal bowel habits (0, nearly normalized; 1, considerably relieved; 2, somewhat relieved; 3, unchanged; and 4, worsened).

Symptoms related to the chief complaint were clarified by an investigator at an interview. The investigator scored the most bothersome IBS symptoms the patient had (nothing, abdominal pain/discomfort, stool form, stool frequency, urgency, feeling of incomplete evacuation, and others) as symptoms of the chief complaint at the week 0, 4, 8 and 12 (or at discontinuation) visits. The investigator also scored any improvement in symptoms of the chief complaint the patients had before administration compared to the baseline period on a five-point ordinate scale (0, completely relieved; 1, considerably relieved; 2, somewhat relieved; 3, unchanged; and 4, worsened) at the week 4, 8 and 12 (or at discontinuation) visits.

### Efficacy and safety endpoints

To explore and examine variables that allow evaluation of “clinically significant improvements, focusing on the patient’s chief complaint,” the chief complaint and its relief by the study drug were assessed in this exploratory study. As an *ad hoc* analysis, patients with scores of 0 (completely relieved) or 1 (considerably relieved) at each evaluation point were defined as responders, with relief of their chief complaint. Patients with missing data were regarded as non-responders. Improvement in stool consistency was also analyzed for the patients with baseline BSFS scores over five, in an *ad hoc* manner. Patients with weekly mean BSFS scores of 3 to 5 during one week of the treatment period and a decrease of one or more points in mean BSFS scores from the baseline period were defined as weekly responders. Patients who were weekly responders for at least two of four weeks in a month were considered monthly responders. If more than two daily scores were missing during any week of the study period, the mean score for that week was defined as missing. Patients with missing mean BSFS scores were regarded as weekly non-responders.

### Statistical analysis

Sample sizes of 60 patients or more (30 patients/group or more) were set based on the feasibility of a post marketing study to explore and examine endpoints rerated to the patient’s chief complaint or IBS severity. Statistical analysis was performed using SAS Drug Development (ver. 3.4) and PC-SAS (ver. 8.2) (SAS Institute Inc., Cary, NC, USA).

Efficacy analyses were performed on the full analysis set (FAS), which was as complete as possible and as close as possible to the intention-to-treat ideal of including all randomized subjects. The FAS included all patients who received at least one dose of the study drug during the treatment period and for whom at least one endpoint could be evaluated. To determine the robustness of the results, primary analyses were performed according to the per-protocol set. Safety analyses were performed for all patients who received at least one dose of the study drug during the treatment period.

Improvement in symptoms of the chief complaint that patients had before administration was scored at each evaluation point. The treatment groups were compared using the Wilcoxon rank sum test with a two-sided significance level of 0.05.

A responder for improvement in symptoms of the chief complaint was defined as a patient with score of 0 or 1 at each evaluation point, with the chi-square test used for *ad hoc* analysis. BSFS was evaluated using the *t*-test. Monthly responder rates for improvement in stool consistency were analyzed in an *ad hoc* manner for patients with baseline BSFS scores over 5, using a chi-square test.

There was no adjustment for multiplicity in this study.

## Results

### Overall study population

Written informed consent was provided by 115 patients. Of these, 17 patients dropped out and 98 patients were randomly allocated to the ramosetron 5 μg (*n* = 47) or the placebo group (*n* = 51) [24]. Ultimately 44 patients in the ramosetron group and 45 patients in the placebo group completed the study. The reasons for discontinuation are shown in the previous report. In this study, one patient in the placebo group who discontinued after randomization by withdrawing consent with no data available was excluded from the FAS used in the efficacy analyses. The decision to exclude this patient from the FAS was taken before unblinding, according to the predefined procedure stipulated in the study protocol.

All the demographic and baseline characteristics used in this study are shown in the previous report [24] and are similar among patients allocated to each group and almost the same as in other ramosetron clinical studies [17, 18]. Duration of disease was 111.5 ± 129.10 months in the ramosetron 5 μg group and 103.9 ± 90.27 months in the placebo group (*P* = 0.738).

### Efficacy

Table 1 shows the symptoms of the chief complaint that patients had before administration. Abdominal pain/discomfort, stool form, and stool frequency were key symptoms for the patients enrolled in this study. The proportion of patients whose chief complaint was abdominal pain/discomfort was 34.0% in the ramosetron 5 μg group and 42.0% in the placebo group. Regarding stool form and stool frequency, key symptoms among bowel habit abnormalities, the respective proportion of patients was 19.1 and 25.5% in the ramosetron 5 μg group and 18.0 and 20.0% in the placebo group.

**Table 1:** Chief complaint that patients had before administration of the study drug

Chief complaint: symptoms before administration	Placebo (*n* = 50)	Ramosetron 5 μg (*n* = 47)
None	0 (0.0%)	0 (0.0%)
Abdominal pain/discomfort	21 (42.0%)	16 (34.0%)
Stool form	9 (18.0%)	9 (19.1%)
Stool frequency	10 (20.0%)	12 (25.5%)
Urgency	6 (12.0%)	7 (14.9%)
Feelings of incomplete evacuation	4 (8.0%)	3 (6.4%)
Others	0 (0.0%)	0 (0.0%)

**Fig. 1:**
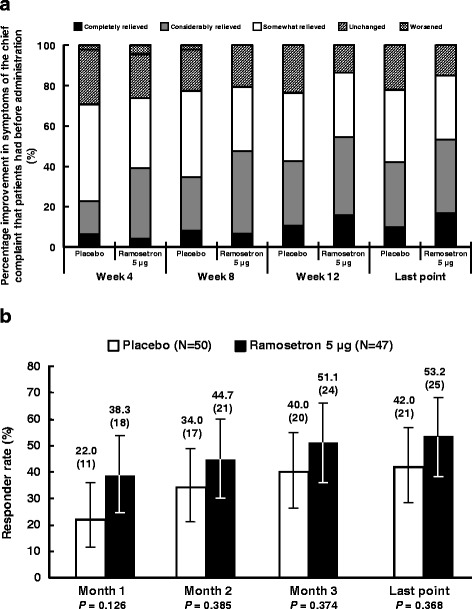
Improvement in symptoms of the chief complaint that patients had before administration of the study drug. **a** Improvement in symptoms of the chief complaint that patients had before administration of the study drug. **b** Responder rate for improvement in symptoms of the chief complaint that patients had before administration of the study drug. Height: responder rate (%). Error bar: 95% CI. *P* values were calculated using the *χ* ^2^-test

Improvement in the symptoms of the chief complaint that patients had before administration was assessed on a five-point ordinate scale at every visit (Fig. 1a). Patients with scores of 0 (completely relieved) or 1 (considerably relieved) at each evaluation point were defined as responders (Fig. 1b. *ad hoc* analysis). Responder rates for improvement in the symptoms of the chief complaint that patients had before administration were 53.2% (95% confidence interval [CI], 38.1–67.9 at the last point) in the ramosetron 5 μg group and 42.0% (95% CI, 28.2–56.8 at the last point, *P* = 0.368) in placebo. The difference between placebo and ramosetron was over 10% at all evaluation points.

**Fig. 2:**
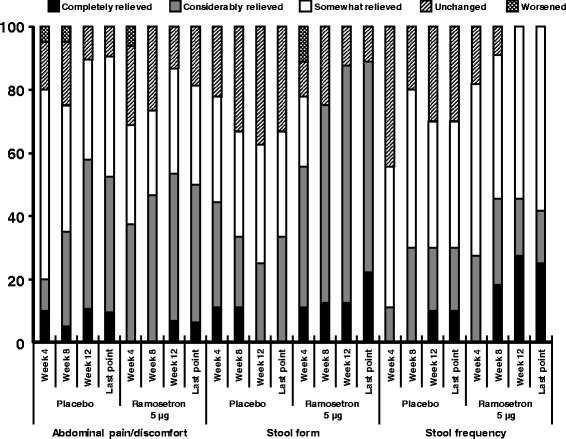
Improvement in symptoms of each chief complaint that patients had before administration of the study drug

Figure 2 shows improvement in the symptoms of each chief complaint that patients had before administration. Regarding stool consistency, the number of patients who had completely relieved or considerably relieved symptoms in the ramosetron 5 μg group increased in a time-dependent manner. Almost all patients showed completely relieved (12.5%) or considerably relieved (75%) symptoms in relation to stool consistency in the ramosetron 5 μg group at Week 12. The difference between ramosetron and placebo was greatest with respect to stool consistency. Improvement in the ramosetron 5 μg group compared to placebo was also observed with respect to stool frequency at all evaluation points. Among patients who had abdominal pain/discomfort as a symptom of their chief complaint before administration, patients in the ramosetron 5 μg group showed numerous improvements at Weeks 4 and 8 compared to patients in the placebo group with the same symptoms, however, the difference between ramosetron and placebo was not clear at Week 12 and at the last evaluation point.

**Fig. 3:**
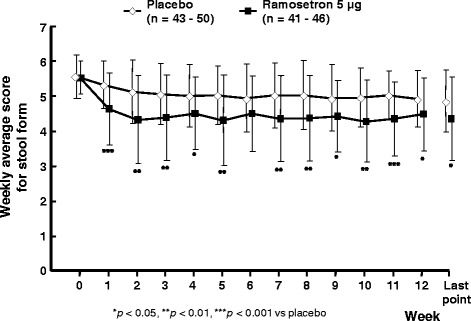
Weekly changes in BSFS scores. *Line graph*: means ± standard deviation. *P* values were calculated using the *χ* ^2^-test, as follows: ***P < 0.001, **P < 0.01, and *P < 0.05

BSFS scores were significantly lower in the ramosetron 5 μg group (4.36 ± 1.195 at the last point) than in the placebo group (4.85 ± 0.890 at the last point, *P* = 0.027) throughout the treatment period, except at week 6 (Fig. 3). No significant difference was observed between the ramosetron 5 μg group and the placebo group regarding changes in the severity of abdominal pain/discomfort and stool frequency from baseline per week (data not shown).

**Fig. 4:**
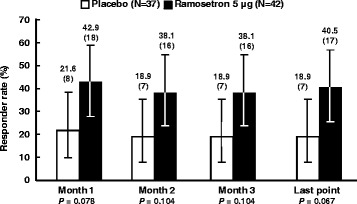
Monthly responder rates for improvement in stool consistency. Height: responder rate (%). Error bar: 95% CI. *P* values were calculated using the *χ* ^2^-test as follows: ***P < 0.001, **P < 0.01, and *P < 0.05

Because stool form was considered to be the most effective symptom for demonstrating if ramosetron brought about a clinically meaningful improvement, monthly responder rates for improvement in stool consistency were analyzed *ad hoc* for patients with baseline BSFS scores over 5 (Fig. 4). Responder rates for improvement in stool consistency were 40.5% (95% CI, 25.6–56.7 at the last point) in the ramosetron 5 μg group and 18.9% (95% CI, 8.0–35.2 at the last point, *P* = 0.067) in the placebo group. The difference between placebo and ramosetron was over 19% at all evaluation points.

**Fig. 5:**
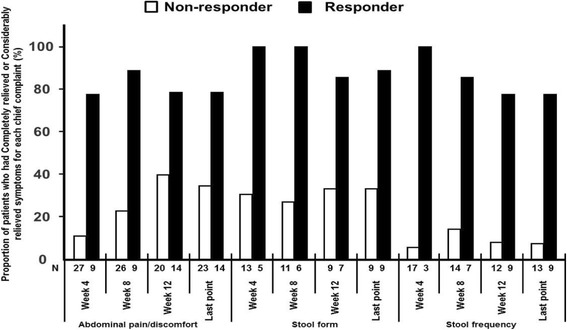
Relationship between improvement in chief compliant and global assessment. Improvement in chief compliant that patients had before administration was compared between responders and non-responders for global assessment of relief of overall IBS symptoms

Improvement in the chief compliant that patients had before administration was compared by responder/non-responder for global assessment of relief of overall IBS symptoms (Fig. 5). Regarding stool consistency, patients who reported that they were completely relieved or considerably relieved in the improvement of chief complaint were more numerous in the responder group on global assessment compared to the non-responder group (8/9, 88.9% vs 3/9, 33.3% at the last point). The same results were observed for abdominal pain/discomfort (11/14, 78.6% vs 8/23, 34.8% at the last point) and stool frequency (7/9, 77.8% vs 1/13, 7.7% at the last point).

### Safety

The incidence of adverse events is shown in Table 2. The incidence of “hard stool” was higher in the ramosetron 5 μg group (19.1%) than in the placebo group (5.9%), which was considered to be caused by the pharmacological action of ramosetron. Constipation was only observed in the placebo group (3.9%). All hard stool and constipation were assessed as drug-related adverse events, except for one patient in placebo with hard stool. All events including constipation and hard stool observed in this study were mild and improved quickly. There was no occurrence of ischemic colitis or serious adverse events.

**Table 2:** Incidence of adverse events

Event	Placebo (*n* = 51)	Ramosetron 5 μg (*n* = 47)
All adverse events	20 (39.2%)	27 (57.4%)
Gastrointestinal disorders	8 (15.7%)	13 (27.7%)
Abdominal discomfort	0 (0.0%)	2 (4.3%)
Constipation	2 (3.9%)	0 (0.0%)
Hard stool	3 (5.9%)	9 (19.1%)
Nausea	2 (3.9%)	0 (0.0%)
Infections and infestations	4 (7.8%)	5 (10.6%)
Nasopharyngitis	4 (7.8%)	3 (6.4%)
Gastroenteritis	0 (0.0%)	2 (4.3%)
Hepatobiliary disorders	2 (3.9%)	2 (4.3%)
Hepatic function abnormal	2 (3.9%)	1 (2.1%)
Skin and subcutaneous tissue disorder disorders	2 (3.9%)	3 (6.4%)
Dermatitis contact	1 (2.0%)	2 (4.3%)

Data are expressed as number (%). Events with an incidence of ≥ 3% in any of the groups are listed

## Discussion

Because IBS is a syndrome, most previous studies to develop agents for IBS have used global assessments as primary endpoints, as these allow patients to assess the improvement of overall symptoms of IBS [17–21, 26, 27]. Individual symptoms of IBS were assessed as secondary endpoints. The most bothersome IBS symptoms reported in clinical trials of alosetron were abdominal pain and urgency [28, 29]. However, there were no data regarding the chief complaint in previous clinical trials of ramosetron. In this study, to evaluate “clinically meaningful improvements” that focus on the patient’s chief complaint, IBS-D patients enrolled in this study were asked about their most bothersome IBS symptoms and improvement of those symptoms at every visit. IBS is characterized by two major IBS symptoms, abdominal pain/discomfort and abnormal bowel habits. Regarding the chief complaint that patients had before administration, 34.0% of the patients in the ramosetron 5 μg group and 42.0% of the patients in the placebo group reported abdominal pain/discomfort. The remaining patients complained of abnormal bowel habits, including abnormal stool form, increased stool frequency, defecation urgency, and a feeling of incomplete evacuation. Of these, the highest proportions were related to stool form (19.1% in ramosetron 5 μg group and 18.0% in placebo group) and stool frequency (25.5, 20.0%, respectively). Stool form and stool frequency were thus considered to be the most important chief complaints among the bowel habit abnormalities. When compared to placebo, the greatest improvement in symptoms of the chief complaint that patients had before administration of ramosetron 5 μg group was shown in stool consistency. This result was consistent with the finding that BSFS scores were significantly lower in the ramosetron 5 μg group than in the placebo group.

We therefore found that ramosetron acted most effectively on stool consistency. Stool consistency correlates with colonic transit time [30] and can be a good indicator of bowel function. In rats, ramosetron also clearly reduced stress-induced diarrhea and accelerated defecation caused by corticotropin-releasing hormone [14, 15]. To show how ramosetron is effective for individual IBS symptoms, focusing on stool consistency was considered to be acceptable in light of the drug’s pharmacological mechanism. However, if the effect of ramosetron on stool consistency is excessive, it leads to constipation. In developing agents to treat IBS-D, it is insufficient to only compare the change in stool form from baseline between ramosetron and placebo. It was considered important to define a clinically meaningful improvement in stool consistency as well. BSFS scores of 3 to 5 are recognized as normal stool form in the Rome III criteria. Therefore, patients with weekly mean BSFS scores of 3 to 5 during one week of the treatment period and a decrease of one or more points in mean BSFS scores from the baseline period were defined as weekly responders. Patients who were weekly responders for at least two of four weeks in one month were defined as monthly responders. We thus defined monthly responder rates in respect to improvement in stool consistency and revealed greater responder rates in the ramosetron 5 μg group compared to placebo group (40.5% vs 18.9% at the last point, *P* = 0.067), by *ad hoc* analysis. Then, we conducted a randomized, placebo-controlled study from October 2010 to August 2011 with male IBS-D patients to evaluate the prospective effect of ramosetron with the improvement of stool consistency as the primary endpoint. A statistically significant improvement in stool consistency was shown in the ramosetron group compared to the placebo group [26].

Because IBS is a syndrome that includes multiple lower gastrointestinal symptoms, it is not known whether patients are satisfied by an improvement in just one universal symptom. As a result, it is considered to be important to assess the improvement of overall IBS symptoms using global assessment during a clinical study of ramosetron. Moreover, the result that global assessment was correlated with improvement in the Japanese version IBS severity index suggests that global assessments reflect improvement of symptom severity in patients with IBS-D [24]. In a phase III study involving female IBS-D patients, the co-primary endpoints of monthly responder rate for global assessment of relief of overall IBS symptoms and improvement in stool consistency were used to evaluate the efficacy of ramosetron. The results showed that efficacy in the ramosetron group was significantly superior to that in the placebo group for both primary endpoints [20]. BSFS scores were also significantly lower in the ramosetron 2.5 μg group than in the placebo group.

Although it was unclear that an improvement of one chief complaint influenced the other IBS symptoms of patients, our results suggest that improvement in the chief complaint the patient had before administration was more frequent in the responder group in global assessment compared to the non-responder group. Improvement of each IBS symptom seems to be related to improvement of overall symptoms. These relationships were obtained not only for stool form, but also for abdominal pain/discomfort and stool frequency. In another study with larger patient numbers, ramosetron showed a statistically significant improvement in the severity of abdominal pain/discomfort and stool frequency compared to placebo at some evaluation points [20, 26]. Ramosetron was suggested to improve abdominal pain/discomfort and stool frequency as well. Stress is considered to induce abnormal GI motility via efferent nerves, which in turn worsens the stress response by stimulating afferent nerves resulting visceral hypersensitivity. Another clinical trial with ramosetron showed that overall scores, dysphoria, interference with activity, and food avoidance included in IBS-QOL, disease specific health-related QOL were significantly improved by ramosetron treatment (5 μg for male and 2.5 μg for female) compared to placebo [20, 26]. Thus, ramosetron is likely to induce a clinical improvement in the stress-related disturbance of brain-gut interactions by reducing exaggerated pain sensitivity and colonic motility in the gut.

The Japanese Society of Gastroenterology developed evidence-based diagnostic and therapeutic algorithms for the Japanese IBS guidelines [31]. In their report, 5-HT3 antagonists should be used for IBS-D at step 1 therapy. Step 2 therapy begins by evaluating the role of psychosocial stress on each IBS patient. Almost all of the sites in this study were primary care sites two patients had current evidence of anxiety neurosis and no patients had depression. This number of patients was within our expectations, and the severity of the anxiety was not severe. In the United Kingdom, 58% of the patients with IBS are diagnosed by general practitioners, with up to 29% of the patients referred to a specialist when primary care management proves to be difficult or ineffective [32]. Koloski et al. reported that the central nervous system and gut interact bidirectionally in functional gastrointestinal disorders, including IBS and functional dyspepsia [33]. Ramosetron 5 μg for male patients with IBS-D showed significant improvement compared to placebo in weekly responders for global assessment of relief of overall IBS symptoms and improvement in stool consistency at week one, with the improvement sustained throughout the treatment period [26]. The same results were shown for ramosetron 2.5 μg for female patients with IBS-D [20]. It is considered to be important to improve gastrointestinal disorders before the symptoms of patients move to Step 2 therapy. Those patients who have moderate severity with anxiety and/or depression are not expected to response to gut targeted pharmacotherapy.

Ramosetron improved stool consistency, but also occasionally triggered constipation and hard stool. Actually, the incidence of hard stool in the ramosetron 5 μg group (9/47, 19.1%) was higher than that of the placebo group (3/51, 5.9%) in this study. These adverse events were also observed, with a statistically significant difference between ramosetron and placebo in a study with a larger sample. The incidence of hard stool was 12/147 (8.2%) in the ramosetron 5 μg group and 2/149 (1.3%, *P* = 0.006) in the placebo group for male patients [26]. Additionally, for female patients the incidences of constipation and hard stool were significantly higher in the ramosetron 2.5 μg group (32/292, 11.0 and 66/292, 22.6%, respectively) than in the placebo group (13/284, 4.6%, *P* = 0.005; and 16/284, 5.6%, *P* < 0.001, respectively) [20]. These were considered to be caused by the pharmacological action of ramosetron.

This post marketing study was a pilot study and has some limitations. First, the chief complaint was obtained at an interview and recorded by the investigator. No patients replied about symptoms other than those that were pre-listed. Bloating has recently been reported to be the most troublesome symptom in IBS patients [6, 34]. In previous studies of alosetron conducted in patients with IBS-D, about 10% of the patients reported bloating as their most bothersome IBS symptom [28, 29]. However, bloating was not captured as a chief complaint on eliciting symptoms in this study. Second, the population of this study was limited to male patients.

The United States Food and Drug Administration (FDA) proposed a study design for clinical trials focused on IBS that would assist the pharmaceutical industry and investigators who are developing drugs [35]. These guidelines suggest the use of abdominal pain and stool consistency as co-primary endpoints for IBS-D. They also recommend the development of multi-item patient-reported outcome (PRO) instruments that can capture clinically important signs and symptoms of the IBS target population (e.g., IBS-C or IBS-D). Some PRO measurements are under construction in the study of IBS [27, 36] in accordance with FDA guidance for PRO [37]. In its PRO guidance, FDA recommends that acceptable PRO must be couched in an explicit and evidence based conceptual framework. In the future, PRO measurements that include validated assessment of multiple chief complaints might be available in clinical trials related to IBS.

## Conclusion

Ramosetron acted most effectively on stool consistency. Improvement in stool consistency is considered to be a clinically meaningful endpoint in showing how ramosetron was effective for individual IBS symptoms. We found that the monthly responder rate for improvement in stool consistency can be used as a co-primary endpoint, together with global assessment.

## Abbreviations

5-HT: 5-hydroxytryptamine
5-HT3: 5-hydroxytryptamine 3
95% CI: 95% confidence interval
BSFS: Bristol Stool Form Scale
FAS: Full analysis set
FDA: Food and drug administration
GI: Gastrointestinal
IBS: Irritable bowel syndrome
IBS-C: Irritable bowel syndrome with constipation
IBS-D: Irritable bowel syndrome with diarrhea
PRO: Patient-reported outcome
QOL: Quality of life
